# Transcriptome assemblies of *Cynanchum wilfordii* and its related species (Apocynaceae)

**DOI:** 10.1186/s13104-026-07753-2

**Published:** 2026-03-13

**Authors:** Mi-Jeong Yoo, Ji-Eun Choi, Jina Lim, Byoung Yoon Lee, Chae Eun Lim

**Affiliations:** 1 Department of Biology, Clarkson University, Potsdam, NY 13699 USA; 2 National Institute of Biological Resources, 42 Hwangyeong-ro, Seo-gu, Incheon, 22689 Republic of Korea; 3 Department of Biology Education, Kongju National University, Gongju-si, Chungcheongnam-do 32588 Republic of Korea

**Keywords:** RNA-seq, Transcriptome, De novo assembly, *Cynanchum wilfordii*

## Abstract

**Objectives:**

*Cynanchum wilfordii* is an important medicinal plant whose authenticated use is complicated by frequent adulteration with closely related species. Because genetic divergence among *Cynanchum* taxa is low, currently available molecular markers provide limited resolution. To address this challenge, we generated and analyzed transcriptome assemblies for *C. wilfordii* and related species, with the aim of identifying informative nuclear genes applicable to phylogenetic analyses and molecular marker development.

**Data description:**

Four RNA-seq libraries were generated from young leaf tissues of *C. wilfordii*, *C. chinense*, *C. rostellatum*, and *Vincetoxicum floribundum*, and sequenced on an Illumina NovaSeq 6000. Raw reads of *C. auriculatum* (re-identified as *C. boudieri*), a close relative of *C. wilfordii*, were also included. Clean reads were assembled *de novo* with Trinity, clustered into unigenes using CD-HIT-EST, and functionally annotated across multiple databases. Open reading frames were predicted with TransDecoder, and assembly quality was evaluated with BUSCO, using predicted mRNA transcripts from the *Asclepias syriaca* genome for comparison. Single-copy orthologues identified by OrthoFinder across six species were analyzed phylogenetically. These transcriptome data provide resources for identifying single- or low-copy nuclear genes useful in Apocynaceae phylogenetics and for developing molecular markers in *C. wilfordii*.

## Objective

*Cynanchum wilfordii* (Maxim.) Hook. fil., commonly known as swallowwort or Korean swallowwort, has been used in traditional herbal medicine due to its bioactive compounds, such as triterpenoid saponins, flavonoids, and alkaloids. Thus, it has been employed for many health concerns, including the enhancement of immunity and the treatment of musculoskeletal conditions, particularly those related to postmenopausal conditions like osteoporosis [1]. Besides its traditional uses, *C. wilfordii* has attracted attention to Western medicine for its prospective anticancer properties. Studies indicate that extracts from the plant exhibit cytotoxic effects on various cancer cell types. This has prompted further investigation into its potential application in cancer treatment, with current studies exploring its mechanisms of action and therapeutic efficacy [2].

Only the root of *C. wilfordii* is employed in the extraction of desirable chemicals, and it is typically marketed as *C. wilfordii* radix. However, unethical individuals may sell the root tuber of closely related species under the same name. This has necessitated the development of molecular markers capable of effectively distinguishing *C. wilfordii* from its closely related species. However, it should be noted that *C. wilfordii* and its related species demonstrate remarkably low genetic diversity, which raises questions regarding the reliability and effectiveness of current molecular markers for their differentiation [3, 4].

Therefore, in this study, we conducted transcriptome assembly of *C. wilfordii* and its related species. The assembled transcriptomes from this study can serve as valuable resources for identifying common single- or low-copy nuclear genes among *C. wilfordii* and its related species. These identified genes have potential applications in phylogenetic frameworks, allowing for a better understanding of the evolutionary relationships within this group. Furthermore, the identified genes may be utilized in the development of molecular markers, providing resources for genetic and molecular research in *Cynanchum* species.

## Data description

Four RNA-seq libraries were constructed with young leaf tissues of *C. wilfordii*, *C. chinense*, *C. rostellatum*, and *V. floribundum*, and sequenced on an Illumina NovaSeq 6000 as 100 bp paired-end reads. Voucher specimens for these samples—celim1372 for *C. chinense*, LeeJD et al. 19,395 for *C. rostellatum*, LeeJD et al. 19,361, and LeeJD et al. 19,319 for *V. floribundum*—have been deposited in the herbarium of the National Institute of Biological Resources (NIBR), Incheon, South Korea. Their collection information is provided in Data file 1 (Table 1), along with methodological details of the RNA sequencing and data analysis. The raw data of Illumina RNA-seq data were deposited in the NCBI Sequence Read Archive (SRA) database (Data files 2–5, Table 1).

**Table 1:** Overview of data files/data sets

Label	Description of data file	File types (file extension)	Data repository and identifier (DOI or accession number)
Data file 1	Methodology	MS Word file (.docx)	Figshare 10.6084/m9.figshare.30197980 [7]
Data file 2	RNA-seq dataset of *C. wilfordii* obtained in this study	Fastq files (.fastq)	NCBI https://identifiers.org/ncbi/insdc.sra:SRX20755942 [8]
Data file 3	RNA-seq dataset of *C. rostellatum* obtained in this study	Fastq files (.fastq)	NCBI https://identifiers.org/ncbi/insdc.sra:SRX20755943 [9]
Data file 4	RNA-seq dataset of *V. floribundum* obtained in this study	Fastq files (.fastq)	NCBI https://identifiers.org/ncbi/insdc.sra:SRX20755944 [10]
Data file 5	RNA-seq dataset of *C. chinense* obtained in this study	Fastq files (.fastq)	NCBI https://identifiers.org/ncbi/insdc.sra:SRX20755945 [11]
Data file 6	Transcriptome assembly of *C. wilfordii*	Fasta files (.fasta)	Figshare 10.6084/m9.figshare.30198115 [12]
Data file 7	Transcriptome assembly of *C. boudieri*	Fasta files (.fasta)	Figshare 10.6084/m9.figshare.30330022 [13]
Data file 8	Transcriptome assembly of *C. chinense*	Fasta files (.fasta)	Figshare 10.6084/m9.figshare.30330226 [14]
Data file 9	Transcriptome assembly of *C. rostellatum*	Fasta files (.fasta)	Figshare 10.6084/m9.figshare.30330238 [15]
Data file 10	Transcriptome assembly of *V. floribundum*	Fasta files (.fasta)	Figshare 10.6084/m9.figshare.30330247 [16]
Data file 11	Summary of transcriptome assembly and annotation	MS Excel file (.xlsx)	Figshare 10.6084/m9.figshare.30198178 [17]
Data file 12	BUSCO assessment results	Image file (.tif)	Figshare 10.6084/m9.figshare.30199906 [18]
Data file 13	Phylogenetic trees	Image file (.tif)	Figshare 10.6084/m9.figshare.30199933 [19]

A total of 223,231,063 paired reads were newly generated, and 219,877,302 reads were used for assembly. As a result, 23,397 (*C. wilfordii*) to 35,658 (*C. boudieri*) unigenes were assembled, which have at least 300 bp of ORF (Data files 6–10, Table 1). Various database search results showed that 44–94% of assembled transcripts were annotated, and eggNOG gave the highest number of genes annotated (Data file 11, Table 1). The annotated unigenes were functionally classified based on the Gene Ontology (GO) database using Blast2GO [5]. The GO terms belonging to biological process, cellular component, and molecular function were listed. For example, GO annotations of *C. wilfordii* assembly resulted in 14.2%, 14.6%, and 13.5% of unigenes assigning to BP, CC, and MF, respectively, and other species showed similar results.

The completeness of assembled transcriptomes was evaluated with BUSCO, and four newly assembled transcriptomes exhibited similar completeness (complete (C) and single-copy (S)) (83–87%), which is comparable to that of *A. syrica* (83%) (Data file 12, Table 1). However, transcriptome of *C. boudieri* showed lower completeness (69%) compared to others as well as the high number of duplicated (D) transcripts. This result indicates this individual may be a polyploid species because both diploid and polyploid *Cynanchum* species had been reported in Japan [6]. However, it is not clear whether this low completeness and high number of duplicated transcripts reflect actual biological factors, such as polyploidy, or assembly artifacts.

Putative orthologs search was performed with OrthoFinder, and as a result 3,623 single-copy orthologues were identified from six species. Comparison of trees from all orthologues, single orthologues, and RNA-binding KH domain-containing protein RCF sequences showed differences in the position of *C. chinense* and *C. rostellatum* (Data file 13, Table 1). The low support values and short branch length (Data file 13, Table 1) suggest that the low genetic diversity among *Cynanchum* species. Although a gene concordance factor of 64% indicates that a majority of loci support the *Cynanchum* clade, appreciable gene tree discordance remains, suggesting heterogeneous evolutionary histories among loci.

## Limitations

The transcriptome assemblies were derived from RNA extracted from young leaf tissues of a single individual, thereby representing the transcripts expressed in leaves at one specific time point for each species. Moreover, developmental stages were not coordinated among the five species, so certain assembled transcripts may be species-specific, which limits the recovery of shared single-copy orthologues. Thus, the single-copy orthologues identified here do not constitute the whole set for the investigated taxa.

## Data Availability

The data described in this Data Note can be freely and openly accessed in the NCBI and figshare databases. The raw reads have been deposited in the SRA database of NCBI under accession number PRJNA986070. The assembled transcriptomes and the other data files are available in the figshare database. Please see Table 1 and references [7–12] for details and links to the data.

## Abbreviations

NCBI: National Center for Biotechnology Information
SRA: Sequence Read Archive
GO: Gene Ontology
